# Oral health‐related quality of life among young adults with cleft in northern Finland

**DOI:** 10.1002/cre2.284

**Published:** 2020-05-12

**Authors:** Mirjami Corcoran, Saujanya Karki, Virpi Harila, Helvi Kyngäs, Anni Luoto, Leena P. Ylikontiola, George K. Sándor, Vuokko Anttonen

**Affiliations:** ^1^ Research Unit of Oral Health Sciences University of Oulu Oulu Finland; ^2^ Medical Research Centre Oulu Oulu University Hospital and University of Oulu Oulu Finland; ^3^ Research Unit of Nursing Science and Health Management University of Oulu and University Hospital of Oulu Oulu Finland

**Keywords:** cleft lip, cleft palate, oral health, quality of life

## Abstract

**Objectives:**

This study aimed to examine Oral Health‐related Quality of Life (OHRQoL) among patients with cleft lip with or without palate (CLP) at their final scheduled follow‐up visit at the age of 18 years. Another aim was to investigate the motivation to attend multiple follow‐up appointments and the satisfaction with care given by the cleft team using inductive qualitative analysis.

**Methods:**

This cross‐sectional study was conducted among the cohort of children born with CLP who had undergone treatment at the Oulu University Hospital Cleft Lip and Palate Centre, in northern Finland since 1995. OHRQoL was assessed using the validated Finnish version of the short form of the Oral Health Impact Profile (OHIP‐14). In addition to the OHIP‐14, two open‐ended questions were also included. These questions investigated the experience of each participant concerning their motivation to attend the Oulu University Hospital Cleft Lip and Palate Centre to receive complex treatments, and their satisfaction with care provided by the cleft team. Results were presented as proportions, means, and *SD*. Inductive content analysis method was performed for analysis of the open‐ended questions.

**Results:**

A total of 63 patients with CLP participated in this study. More than half of the participants had cleft palate. More than half of the participants reported an impact on OHRQoL (OHIP‐14 score ≥ 3). All the participants with bilateral cleft lip and palate, three fourths of the participants with unilateral cleft lip and palate, and half of the participants with cleft palate reported impact on OHRQoL. Inductive content analysis showed that one fourth of the participants reported a good outcome as a motivation to attend cleft center despite of complex procedures. All the participants reported their appreciation of the cleft team.

**Conclusions:**

Despite the comprehensive treatment received by the patients born with a CLP, they still experienced lower OHRQoL, especially physical pain and psychological discomfort were more pronounced. However, good outcome, support, and oral health care being a normal routine were the motivating factors to attend a long and demanding oral health care regimen.

## 1 INTRODUCTION

Cleft lip with or without palate (CLP) is one of the most common congenital craniofacial anomalies (Klassen et al., 2012). According to the global burden of disease study, the recent worldwide incidence of orofacial clefts was 195,500 from 1990 to 2017 (James et al., 2018). The prevalence of cleft palate significantly varies within the European countries, and a higher prevalence was reported in Finland compared to other European countries (Calzolari, Bianchi, Rubini, Ritvanen, & Neville, 2004). It was estimated to be 2.56 cases per 1,000 live births and abortions in Finland during the years 1993–1996.(Calzolari et al., 2004). In northern Finland, almost two thirds of the children born with a cleft had only a cleft palate during 1998–2011 whereas approximately two fifths had cleft lip and palate, and one tenth had cleft lip (Lithovius, Ylikontiola, Harila, & Sándor, 2014).

Children born with a cleft are often affected by speech problems, deviations in facial morphology, and a variety of anomalies in dentition (Klassen et al., 2012; Lithovius et al., 2014). They may also encounter short‐ or long‐term challenges including treatment burden from ear infections and hearing problems as well as associated psychological issues (Stock, Feragen, & Rumsey, 2015; Thomson & Broder, 2018). They often need multidisciplinary treatment from childhood to adulthood and even lifelong care that may require complex surgical procedures, orthodontic treatment, speech therapy, and psychological counseling (Stock et al., 2015). These treatment modalities are aimed toward normal physical functioning as well as for the psychological and social well‐being of the individual.

Oral Health‐related Quality of Life (OHRQoL) is a patient‐reported outcome measure for evaluating the functional, emotional, and psychosocial aspects of oral health (Locker & Allen, 2007). In general, OHRQoL is a subjective component of oral health. A systematic review and meta‐analysis reported that the presence of CLP negatively affects the OHRQoL (de Queiroz Herkrath, Rebelo, & Vettore, 2015). Children with CLP of different ages have reported lower OHRQoL than those without a cleft (Foo, Sampson, Roberts, Jamieson, & David, 2012; Kortelainen et al., 2016). Similarly, children with CLP also have a higher impact on OHRQoL compared to those with oral diseases like dental caries and/or malocclusion (Jokovic et al., 2002; Khoun, Malden, & Turton, 2018).

It is important to evaluate OHRQoL among individuals born with CLP throughout their lifetime. Moreover, the inclusion of qualitative assessments for evaluating the treatment burden and satisfaction with care among the cohort can be valuable. Therefore, this study aimed to examine OHRQoL among patients with CLP at their final scheduled follow‐up visit at the age of 18 years. Another aim was to investigate the motivation to attend multiple follow‐ups and satisfaction with the care given by the cleft team using inductive qualitative analysis.

The authors hypothesized that CLP has an impact on OHRQoL in this study population, and there is no difference between gender and types of clefts. A second hypothesis is that the treatment burden decreased motivation for dental attendance.

## 2 METHODS

This cross‐sectional study was conducted among the cohort of children born with CLP who had undergone treatment at the Oulu University Hospital Cleft Lip and Palate Centre, in northern Finland since 1995. The center's cleft team consists of a group of health care professionals including surgeons, orthodontists, pediatric dentists, ENT specialists, psychiatrists, foniatrists, speech therapists, and nurses. The cleft team conducts and provides all comprehensive treatments.

Ethical approval for this study was obtained from the Hospital District of Northern Ostrobothnia (permission number 10/2012). This study was voluntary and verbal consent was received from the study participants. A total of 63 out of 64 individuals at their last follow‐up visit at the age of 18 years participated in the survey. Data were collected between 2015 and 2019.

Background information regarding gender, date of birth, and type of cleft were included in the questionnaire without any personal identification. One of the nurses delivered the questionnaire to all the 18‐years‐old patients at their last scheduled follow‐up visit at the cleft clinic of Oulu University Hospital. Participants were asked to complete the form by themselves in a self‐administered fashion. However, in the case of disabled children their parents were asked to assist. After completion of questionnaire, the participants were asked to deposit the forms in a sealed container.

### 2.1 Oral health‐related quality of life

OHRQoL was assessed using the validated Finnish version of the short form of the Oral Health Impact Profile (OHIP‐14) (Lahti et al., 2008). The OHIP‐14 consists of seven domains (*Functional Limitation, Physical Pain, Psychological Discomfort, Physical Disability, Psychological Disability, Social Disability, and Handicap*) and each domain includes two questions. For each question, participants were given a reference period of 1 month. The response options for each question were made using a Likert scale as follows: very often, fairly often, sometimes, very seldom, never, and do not know (Lahti, Suominen‐Taipale, & Hausen, 2008).

In addition to the OHIP‐14, two open‐ended questions were also included. These questions investigated the experience of each participant concerning their motivation to attend the Oulu University Hospital Cleft Lip and Palate Centre to receive complex treatments, and their satisfaction with care provided by the cleft team (a) *What has given you strength or motivation to go through your procedures involving the mouth, jaws, or teeth?* (b) *What message do you have for the Hospital*'*s Cleft team*. The entire questionnaire was self‐administered.

### 2.2 Statistical analyses

All data were transferred into an electronic database for analyses using the SPSS software (IBM SPSS Statistics for Windows, version 24.0; IBM Corp., Armonk, NY). For data entry, the responses were coded as follows: “do not know” and “never” = 0, “very seldom” = 1, “sometimes” = 2, “fairly often” = 3, and “very often” = 4. Using the additive scoring method, the score for each domain and total OHIP‐14 score were calculated. The maximum score for each domain ranged from 0 to 8, and that for total OHIP‐14 score ranged from 0 to 56.

Participant's characteristics were categorized and presented as proportions, means, and *standard deviation* (*SD*). The cleft group were categorized as a cleft involving the palate (*cleft palate and a bony defect of the hard palate*) and cleft involving lip (*cleft lip, unilateral cleft lip and palate, and bilateral cleft lip and palate*). Based on the median split, the OHRQoL was categorized into “no impact” and “impact.” As the median score was 3, a total OHIP‐14 score ≤ 2 was considered as “no impact on OHRQoL” and a total OHIP‐14 score ≥ 3 was considered as having an “impact on OHRQoL”. The difference in proportion between groups was compared using the Chi‐square test. The difference in means between groups was compared using the Student *t* test. For all analyses, *p <* .05 was considered statistically significant.

**Figure 1:**
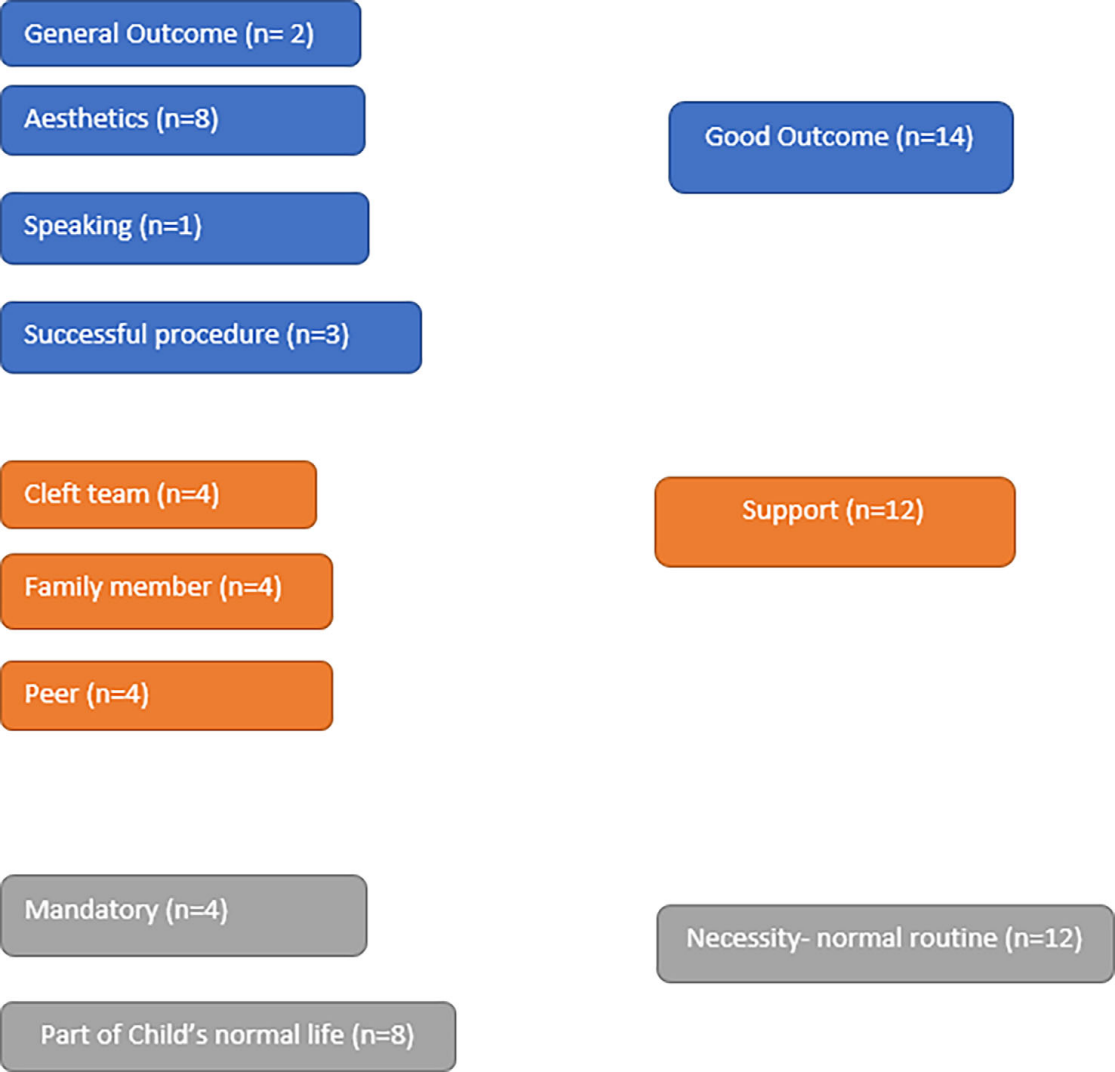
Motivation to attend for multiple follow‐ups

**Figure 2:**
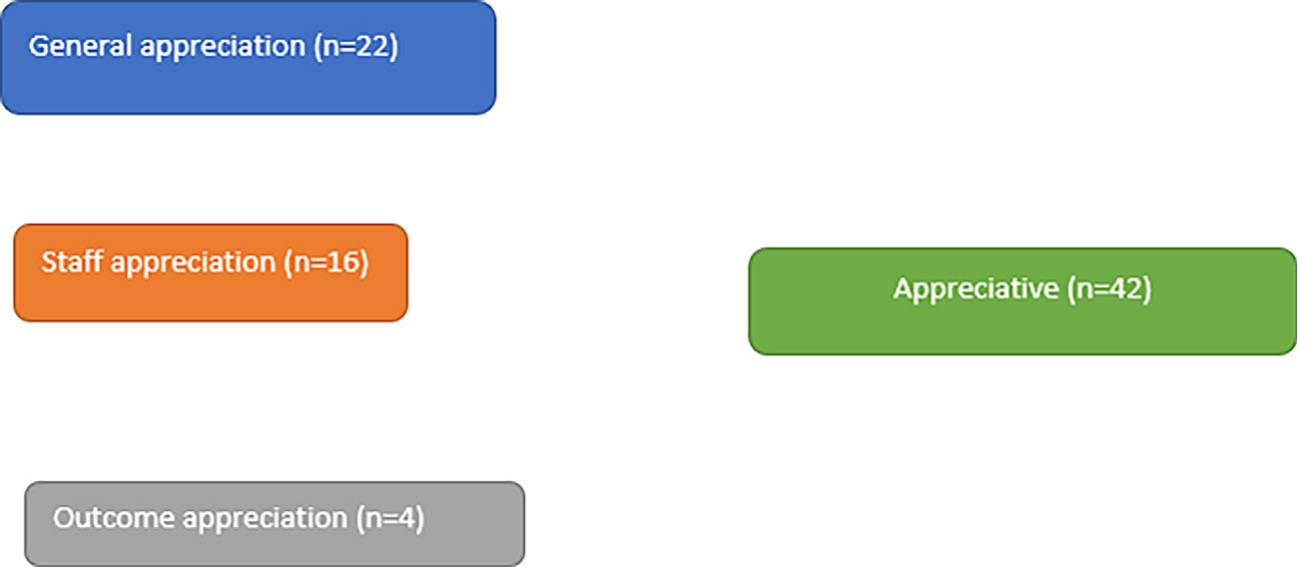
Satisfaction of participants with care provided by the cleft team

For the analysis of the open‐ended questions, an inductive analysis process proposed by Elo and Kyngäs (2008) was performed. In brief, initially, a unit analysis was performed by detailing the responses from participants. This was followed by opening codes and comparing them. Then, the open codes with similar content were sub‐categorized. They were again combined to create generic categories (Figures 1 and 2). The results of qualitative analysis were quantified by calculating how many participants mentioned the issue of subcategory and generic category.

## 3 RESULTS

The study population here was slightly dominated by girls (*n* = 40). More than half of the participants had cleft palate (*n* = 33). Most boys had cleft lip (60.0%) and most of the girls had cleft palate (72.7%) (Table 1).

**Table 1:** Distribution (%) of study participants according to the types of cleft stratified by gender

Types of cleft	Gender (%)	
	Boys (*n* = 23)	Girls (*n* = 40)
Cleft lip (*n* = 5)	60.0	40.0
Unilateral cleft lip and palate (*n* = 13)	30.8	69.2
Bilateral cleft lip and palate (*n* = 2)	100.0	0.0
Cleft palate (*n* = 33)	27.3	72.7
Bony defect of the hard palate (*n* = 8)	62.5	37.5
Total (*n* = 63)	37.7	62.3

Two thirds of the participants reported having physical pain and almost half of them reported psychological discomfort during the past month. The mean (*SD*) OHIP‐14 score was 5.1 (5.7). The participants with a cleft including lip reported poorer OHRQoL than their counterparts with cleft palate alone, particularly concerning psychological discomfort and psychological disability (Table 2).

**Table 2:** Mean (*SD*) OHIP‐14 scores and its domains by gender and types of cleft

Domains of OHIP‐14	Mean (*SD*)	Gender		*p* value	Types of cleft		*p* value
		Boys Mean (*SD*)	Girls Mean (*SD*)		Cleft including lip Mean (*SD*)	Cleft palate Mean (SD)	
Functional limitation	0.71 (1.10)	0.83 (1.34)	0.65 (0.95)	.58	0.65 (1.04)	0.78 (1.15)	.66
Physical pain	1.29 (1.37)	1.26 (1.25)	1.30 (1.45)	.91	1.45 (1.19)	1.27 (1.47)	.61
Psychological discomfort	1.16 (1.62)	1.30 (1.64)	1.08 (1.63)	.59	1.55 (1.85)	1.02 (1.51)	.28
Physical disability	0.32 (0.89)	0.17 (0.49)	0.40 (1.06)	.25	0.15 (0.37)	0.41 (1.07)	.16
Psychological disability	0.86 (1.39)	0.70 (1.26)	0.95 (1.47)	.47	1.25 (1.45)	0.68 (1.37)	.15
Social disability	0.38 (0.87)	0.35 (0.78)	0.40 (0.93)	.81	0.45 (1.00)	0.36 (0.83)	.75
Handicap	0.41 (0.98)	0.30 (0.64)	0.48 (1.09)	.47	0.55 (0.83)	0.37 (1.07)	.46
OHIP‐14 score	5.13 (5.72)	4.91 (5.58)	5.25 (5.86)	.82	6.05 (5.32)	4.90 (5.98)	.45

*Note: p* value computed by *t* tests.

Abbreviation: OHIP‐14, Oral Health Impact Profile‐14.

More than half of the participants reported an impact on OHRQoL (OHIP‐14 score ≥ 3). All the participants with bilateral cleft lip and palate, three fourths of the participants with unilateral cleft lip and palate, and half of the participants with cleft palate reported an impact on the OHRQoL (Table 3).

**Table 3:** Distribution of OHIP‐14 categories by types of cleft

Types of cleft	OHIP‐14 category		*p* value
	No impact (OHIP‐14 score ≤ 2)	Impact (OHIP‐14 score ≥ 3)	
Cleft lip (*n* = 5)	60.0	40.0	.110
Unilateral cleft lip and palate (*n* = 13)	23.1	76.9	
Bilateral cleft lip and palate (*n* = 2)	0.0	100.0	
Cleft palate (*n* = 33)	45.5	54.5	
Bony defect of the hard palate (*n* = 8)	75.0	25.0	

*Note: p* value computed by Chi‐square test.

Abbreviation: OHIP‐14, Oral Health Impact Profile‐14.

Figures 1 and 2 depict the inductive content analysis for motivation and satisfaction with care, respectively. Participants were motivated to attend dental care to achieve a good outcome (*n* = 14), support given by family members, support by dental staff and peers (*n* = 12), followed by feeling of dental treatment as necessity‐normal routine (*n* = 12) (Figure 1). The most revealing comment concerning motivation was “*I can see from the photograph that the facial form and characteristics are changing because of the braces*.” Concerning the question on satisfaction with care from the cleft team, general appreciation (*n* = 22) was reported by most of the participants (*n* = 22) followed by appreciation of the staff (*n* = 16) and outcome (4) (Figure 2). The most highlighting one was “*I have been amazed by your professionalism and I have become more and more grateful for what you have done. On behalf of children with the clefts, I say big thank you to your team*.”

## 4 DISCUSSION

This study aimed to evaluate the OHRQoL among the cohort of children born with CPL in northern Finland at the age of 18, in their last scheduled follow‐up at the Oulu University Hospital Cleft Center. Another aim was to investigate their motivation to attend the cleft center through early years to adulthood for complex procedures and their satisfaction with the care provided by the cleft team. Over half of the participants reported having an impact on OHRQoL, mostly physical pain and psychological discomfort. No difference on OHIP‐14 score was observed between genders and the types of clefts. Inductive content analysis showed that one fourth of the participants reported a good outcome as a motivation to attend cleft center despite complex procedures. All the participants reported their appreciation of the cleft team.

One of the strengths of this study is that all the participants were treated by the same cleft team in the tertiary healthcare center from the birth to the adulthood. This was possible because also children with CLP receive free health care up to 18 years including oral health in Finland. Another strength would be inclusion of both quantitative and qualitative approaches in the present study, as recently suggested (Thomson & Broder, 2018). The present study is a descriptive one and there were no comparable groups which can be considered as a limitation of this study. The sample size can be considered small; however, the global prevalence of CLP is relatively low, also in Finland (Calzolari et al., 2004; Lithovius et al., 2014). One limitation of this study was that the usually the most satisfied patients and those who have the more positive attitude toward their condition and treatment they received are the ones who actually participate in the completion of the questionnaire. All the patients reaching the age of 18 years were invited to participate in a blinded fashion in order to minimize the effect of this phenomenon.

In the study by Kortelainen et al. (2016) higher impact on OHRQoL was reported among children with CLP and emotional well‐being was the least reported domain (Kortelainen et al., 2016). However, in this study physical pain and psychological discomfort were the most impacted OHRQoL domains. It can be presumed that the present study participants are more concerned about emotions and psychological aspects of life than the participants in the study by Kortelainen et al. (2016), despite of the same study site (Kortelainen et al., 2016). Participants in this study were in their late adolescent stage and this may be a reason for emotional and psychological concern. Foo et al. (2012) also identified physical function as one of the most impacted quality of life domains which is in agreement with this study (Foo et al., 2012). Additionally, higher OHIP‐14 score among participants with CLP was observed in both studies, however, the study by Foo et al. (2012) had participants from a national survey as a comparison group (Foo et al., 2012).

Children born with CLP present both short‐ and long‐term challenges including psychological issues. This was also confirmed by a qualitative study conducted among adults with CLP (Stock et al., 2015). As mentioned above, most of the participants here also reported having psychological discomfort. Furthermore, comments on psychological distress evaluated during the inductive content analysis supported this aspect. For instance, one comment reflected the psychological discomfort well, “*I am sensitive to talk about my mouth*.” Inductive content analysis is simple, and less text consuming. This method is recommended for dealing with under studied phenomenon (Elo & Kyngäs, 2008). In addition, this method offers a possibility for the patients to express in their own words their experiences, the reasons for motivation and satisfaction. This is most valuable in developing the treatment protocols *per se* and specifically among the patients with CLP.

The motivation reported by the participants to attend long and demanding oral health care treatments were good outcome, support, and oral health care being normal routine. Esthetic outcomes are most highlighted among the good outcomes and should be taken into consideration in clinical care. In addition to support by the family and peers the support by the cleft team is also essential. Oral health care is part of all children's life and those with CLP are not an exception.

It is surprising that all children reported appreciation, especially staff appreciation after possibly painful or discomfort events during the years of dental treatments. This emphasizes good interaction and positive attitude for the team.

Broder et al. (2017) reported that cleft patients receiving surgery had higher Child Oral Health Impact Profile (COHIP) score than their counterparts without a surgical recommendation (Broder, Wilson‐Genderson, & Sischo, 2017). Likewise, children with CLP also had lower OHRQoL than those without cleft in northern Finland (Kortelainen et al., 2016). There were no significant differences in OHRQoL between genders, and types of cleft in the present study. Similar findings were reported in previous studies (Broder, Wilson‐Genderson, & Sischo, 2012; Foo et al., 2012). On the contrary, several studies also reported that females experience lower OHRQoL than males with CLP (Broder et al., 2017; Crepaldi et al., 2019; Eslami, Majidi, Aliakbarian, & Hasanzadeh, 2013). In the present study, when OHIP‐14 was dichotomized, a varied trend was observed. Most of the children with cleft of palate involving lip (unilateral or bilateral) seemed to have high impact on OHRQoL (OHIP‐14 score ≥ 3). This may be due to the fact that children with a cleft involving the palate are more prone to ear infections (Lehtonen et al., 2015). Eslami et al. (2013) found no difference in OHRQoL between unilateral cleft lip and palate and bilateral cleft lip and palate patients which is in concordance to this study (Eslami et al., 2013).

Despite multidisciplinary care, patients with CLP still had problems related to oral health that consequently impacted the quality of life. Patients with CLP can be considered as a special group that requires relatively high‐quality treatment/care. More studies are needed to specify their problems, particularly a mixed‐method approach could be useful (Thomson & Broder, 2018). In addition to adequate operative and oral health care, patients with CLP need psychological counseling until adulthood (Stock et al., 2015). Therefore, clinicians as well as psychologists should continuously aim to improve the quality of life among CLP patients. Further studies are needed to describe a complete picture of OHRQoL among cleft patients that may include clinical indices such as esthetic evaluation and a comparative study with those without CLP can be an option.

In conclusion, more than half of the patients born with a CLP experience lower OHRQoL, especially physical pain and psychological discomfort were more pronounced. However, no significant differences between gender, and types of cleft were found in this study. The motivation to attend a long and demanding oral health care regimens were good outcome, support, and oral health care being normal routine.

## CONFLICT OF INTEREST

The authors declare no conflicts of interest.

## AUTHOR CONTRIBUTIONS

A.L., G.K.S., and V.A. conceived and designed the study. M.C., S.K., and V.A. performed the analysis and interpretation of data and prepared the manuscript. V.H., H.K., A.L., L.P.Y., and G.K.S. reviewed the manuscript and gave their comments.
